# Characterization of isavuconazole pharmacokinetics and pharmacodynamics in a real-life cohort

**DOI:** 10.1093/jacamr/dlag071

**Published:** 2026-05-05

**Authors:** Monia Guidi, Jade Couchepin, Ilana Reinhold, Ilona Kronig, Dionysios Neofytos, Peter Werner Schreiber, Pascal André, Thierry Buclin, Frederic Lamoth

**Affiliations:** Service of Clinical Pharmacology, Lausanne University Hospital and University of Lausanne, Lausanne, Switzerland; Centre for Research and Innovation in Clinical Pharmaceutical Sciences, Lausanne University Hospital and University of Lausanne, Lausanne, Switzerland; Institute of Pharmaceutical Sciences of Western Switzerland, University of Geneva, Geneva, Switzerland; Infectious Diseases Service, Department of Medicine, Lausanne University Hospital and University of Lausanne, Lausanne, Switzerland; Department of Infectious Diseases and Hospital Epidemiology, University Hospital of Zurich and University of Zurich, Zurich, Switzerland; University of Cologne, Faculty of Medicine and University Hospital Cologne, Department I of Internal Medicine, Excellence Center for Medical Mycology, Cologne, Germany; Division of Infectious Diseases, University Hospital of Geneva, Geneva, Switzerland; Division of Infectious Diseases, University Hospital of Geneva, Geneva, Switzerland; Department of Infectious Diseases and Hospital Epidemiology, University Hospital of Zurich and University of Zurich, Zurich, Switzerland; Department of Infectious Diseases and Infection Prevention, Lucerne Cantonal Hospital, Lucerne, Switzerland; Service of Clinical Pharmacology, Lausanne University Hospital and University of Lausanne, Lausanne, Switzerland; Service of Clinical Pharmacology, Lausanne University Hospital and University of Lausanne, Lausanne, Switzerland; Infectious Diseases Service, Department of Medicine, Lausanne University Hospital and University of Lausanne, Lausanne, Switzerland; Institute of Microbiology, Lausanne University Hospital and University of Lausanne, Lausanne, Switzerland

## Abstract

**Objectives:**

Isavuconazole (ISA) is a triazole drug approved for the treatment of invasive aspergillosis and mucormycosis. Due to the relatively low interindividual variability of ISA pharmacokinetics and the lack of defined cutoffs for efficacy and toxicity, therapeutic drug monitoring (TDM) is rarely performed. This study aimed to characterize ISA pharmacokinetics and pharmacodynamics in real-life clinical settings and assess the potential role of TDM.

**Methods:**

We developed and validated a classical population pharmacokinetic (popPK) model using ISA concentrations obtained from patients undergoing TDM at three Swiss university hospitals. Additionally, we performed exploratory pharmacokinetic-pharmacodynamic analyses to investigate associations between ISA trough concentration or AUC, treatment outcomes, and hepatotoxicity using logistic regression models. Model-based simulations allowed assessing the role of TDM in clinical patient management.

**Results:**

A one-compartment model with interindividual variability in clearance best described our data. Among the covariates tested, only body mass index was found to influence the ISA volume of distribution to a clinically significant extent. Exposure-response analyses suggested a trend toward an association with treatment success, but not with toxicity. Model-based simulations show that TDM could increase the proportion of patients within the currently suggested ISA target ranges.

**Conclusion:**

Our results indicate that ISA exposure shows substantial variability and tends to correlate with outcome. This suggests a beneficial role for TDM in optimizing target achievement.

## Introduction

Invasive fungal infections (IFI) are life-threatening infectious complications among patients with impaired immunity, especially those with acute leukaemia and/or allogeneic haematopoietic stem cell transplantation (incidence 5%–10%) or solid-organ transplantation (1%–10%).^1–3^ Triazoles represent the most widely used antifungal drug class. Isavuconazole (ISA) is a new-generation azole with a wide spectrum of antifungal activity and relevant pharmacologic properties, which has been approved for the treatment of invasive aspergillosis and mucormycosis.^4–6^ ISA exhibits several advantages compared to voriconazole and posaconazole, two other anti-mould triazoles. It has been associated with fewer side effects (in particular, less hepatotoxicity), fewer drug-drug interactions (DDI), and a more stable pharmacokinetic (PK) profile with low interindividual variability (IIV).^7–12^

The ratio of AUC over MIC was the pharmacodynamic (PD) parameter associated with success in animal models.^13–16^ In clinical trials, AUC/MIC target attainment could be achieved in >90% and 75%–80% of adult and paediatric patients, respectively.^9,10,17^ The authors considered an AUC ranging from 60 to 233 mg/L/h as a PD target, with the lower limit drawn from observations in the phase 3 SECURE trial and the upper limit from a study evaluating the risk of QT interval prolongation.^12,17,18^ However, previous PD analyses derived from clinical trials failed to demonstrate an association between ISA exposure and outcome or toxicity.^12,19^ For these reasons, unlike voriconazole or posaconazole,^20,21^ ISA is considered usable without routine therapeutic drug monitoring (TDM), although TDM might be indicated in particular circumstances susceptible to increase its PK variability (e.g. treatment failure, DDI, or suspicion of toxicity).^5,22,23^ Because of the difficulty in quantifying AUC/MIC in clinical practice, trough concentrations (i.e. C_min_, measured just before the next dose) are monitored with a suggested target of 2–3 mg/L, based on average concentration ranges observed in clinical trials.^5,7,24^ More recently, an increasing number of cohort studies using ISA in real clinical settings across different populations provided some controversial results about the extent of IIV of ISA exposure and the potential role of TDM.^22,25–30^ While the correlation between ISA exposure and clinical efficacy remains to be demonstrated, some authors have reported an association between C_min_ and toxicity, supporting an upper threshold of about 5 mg/L to minimize the risk of toxicity.^31–33^

We aimed to perform a population pharmacokinetic (popPK) analysis to characterize ISA PK, quantify the associated IIV, and identify contributing factors, using observed concentrations from a previous cohort study of patients having received ISA for the treatment of IFI in three Swiss university hospitals.^34^ Moreover, we conducted exploratory PK-PD analyses to investigate the correlation between ISA exposure and outcome/toxicity.

## Materials and methods

### Patient data

Our analysis included patients treated with ISA for IFI at the university hospitals of Geneva (centre 1), Lausanne (centre 2), and Zürich (centre 3) between January 2017 and December 2020, with at least one concentration measurement.^34^ Patients followed in centres 1 and 2 underwent routine clinical TDM, providing information on drug dosage history (i.e. administration type, amount, and time of dose intakes) and sampling details. In contrast, for patients enrolled in centre 3, the exact time of dose intakes, including the last administration, and sampling could not be retrieved. However, the patients could be confidently assumed to have received ISA at the recommended dosage, and the concentrations could be considered C_min_, as this corresponds to the routine TDM practices for triazoles in this centre. We collected information on demographic characteristics [i.e. body weight (BW), height, sex, age, BMI], and laboratory measurements (i.e. creatinine, liver function tests, and albumin) from the electronic health records of patients followed in centres 1 and 2. Data on ISA therapy start and end or last follow-up, as well as underlying diseases, IFI characteristics, IFI outcomes, and toxicity, were available for patients followed in all three hospitals.

IFIs were classified as proven, probable, or possible according to the European Organisation for Research and Treatment of Cancer (EORTC) and the Mycoses Study Group Education and Research Consortium (MSGERC).^35^ Treatment efficacy was assessed at week 6 from ISA start, based on CT-scan evaluation and according to EORTC-Mycoses Study Group (MSG) consensus criteria, with success defined as complete or partial response.^36^ Hepatotoxicity was defined as an at least 2-fold increase of any liver function test value (alanine aminotransferase, aspartate aminotransferase, alkaline phosphatase, gamma glutamyl transferase, total bilirubin) from the baseline values (at the beginning of ISA therapy).^34^

The study was approved by the local ethics committee for retrospective data reuse (CER-VD, project ID 2020-01641).

### Population pharmacokinetic and pharmacokinetic/pharmacodynamic analyses

Only data from centres 1 and 2, for which complete information on the time of dose administration and sampling could be correctly retrieved, were used for the popPK analysis. This model was then applied to concentrations from the three centres to perform exploratory PK-PD analyses.

We used the non-linear mixed effect modelling (NONMEM, version 7.4.3) software, assisted by Pirana (version 2.9.8) and Pearl speaks NONMEM (PsN, version 4.8.0), for the popPK analysis, and the program language R for data management, visual exploration, and PK-PD analyses.^37–42^

#### PopPK model development

We followed a classical stepwise approach to identify the model that best described ISA data, starting with the estimation of the bioavailability of orally administered ISA relative to the 100% bioavailability of the intravenous dose. Models with an increasing number of compartments and linear elimination were then compared. We evaluated different absorption processes using first-order, zero-order, or Weibull functions, estimating or fixing the associated parameters to literature values^9^ or to values giving a time to peak (T_max_) of 2–4 h as per the manufacturer’s information^43^ because of the paucity of data right after ISA intake. A log-normal distribution was assumed for all the PK parameters, and the association of IIV with each parameter was assessed sequentially. Residual unexplained variability (RUV) was accounted for by comparing additive, proportional, or mixed additive/proportional error models.

We performed covariate analysis according to the standard popPK analysis workflow, with an initial graphical assessment of biologically plausible covariate-parameter relationships, followed by univariate/multivariate forward covariate inclusion and backwards deletion steps. We used allometric scaling (for BW and BMI) or linear relationships to study the association between PK parameters and covariates. We centred and normalized continuous covariates based on the median or typical population value, whichever was more appropriate. We applied the CKD-EPI formula to estimate glomerular filtration rate, which served as both an index of renal function and a general marker of individual health status.^44^ Laboratory values were tested as continuous or categorized variables using twice the upper limit of normality to dichotomize them. We imputed missing covariate values using linear interpolation between two consecutively quantified values.

##### Model selection and evaluation

The likelihood ratio test allowed the comparison of nested models based on the difference in the NONMEM objective function values (ΔOFV), as it approximately follows a χ^2^ distribution with degrees of freedom equal to the number of additional parameters. We applied significance levels of 0.05 (ΔOFV ≤ −3.84) and 0.01 (ΔOFV > 6.63) for model development/forward inclusion and backward deletion steps, respectively. A decrease of at least two in the Akaike Information Criterion (AIC) enabled the selection between non-nested models. Goodness-of-fit (GOF) plots, together with the precision of parameter estimates evaluated through relative standard error (RSE), assisted in model selection. Conditional weighted residuals (CWRES) between −3 and 3 (|CWRES|>3) enabled the identification of outlying data, which were excluded when a reasonable explanation could be identified. Because of data sparseness, a sensitivity analysis removing observations collected earlier than 6 hours post-dose was performed to evaluate their impact on parameter estimations. We used a non-parametric bootstrap (*n* = 2000) to assess final model reliability, and prediction-corrected visual predictive checks (pcVPC) to evaluate its predictive performance.^45^

We applied the base model developed on centres 1 and 2 data to the data collected at centre 3. We used GOF and CWRES to detect outlying data and calculated bias and precision using mean prediction error (MPE) with its 95% confidential interval (CI_95%_), and relative root mean squared error (RMSE) as follows:


(1) MPE(%)=100*[exp(mean(ln(DV)−ln(IPRED))−1]

(2) $$\;RMSE(\text{\%})=100*\left[exp\sqrt{mean{\left(\ln\;(DV)-\ln\;(IPRED)\right)}^{2}}-1\right]$$

#### Exploratory pharmacokinetic-pharmacodynamic analyses

The PK efficacy analysis focused on patients with proven or probable IFIs, who received ISA for more than 4 weeks and for whom ISA was initiated as a first-line therapy or as subsequent-line therapy within 4 weeks from the start of antifungal therapy. Patients who received ISA beyond 4 weeks from IFI diagnosis were excluded from the efficacy analysis, as it was considered a maintenance therapy. Patients with possible IFI were excluded, considering their low/moderate probability of IFI and the role of confounding factors. The PK-toxicity analysis evaluated hepatotoxicity or consequent ISA discontinuation for any reason in the whole population (including all IFI types). Both analyses (efficacy and toxicity) included only patients with at least an ISA measurement within 50 days of the outcome evaluation, to ensure the sample reflected ISA PK.

The base popPK model enabled predicting ISA C_min_ and AUC on the day of study outcome evaluation for patients of all three centres. Of note, the PK indicators were calculated at the time of treatment discontinuation if it occurred before therapeutic response assessment (i.e. date of CT scan) for the PK-efficacy analysis. Additionally, we used PK indicators on the day of the last PK observation for patients not experiencing toxicity in the PK-toxicity study. We employed logistic regression models with a significance level of *P* = 0.05 for the exploratory PK-PD analyses, using log-transformed C_min_ or AUC as explanatory variables. We performed sensitivity analyses by removing the data of patients of centre 3 and those with extremely high/low C_min_ and AUC values, to investigate their impact on the exploratory PK-PD analyses.

#### Model-based simulations and virtual TDM study

We used the final popPK model to generate simulations of 10 000 patients at steady state, each receiving ISA at the standard dose of 200 mg once per day. Individual values of the covariates retained in the model were derived from uniform distributions to mimic the observed ranges in the study population. Individual CL and volume of distribution (V) values were randomly generated from their corresponding log-normal distributions, excluding pairs of values resulting in half-lives outside the range observed in our patient population to ensure plausibility.

We calculated the percentage of virtual subjects whose individual true C_min_ (IPRED_TRUE_) and AUC_TRUE_, estimated as DOSE/CL, fell within the recommended therapeutic ranges for ISA TDM. We then applied a simple TDM strategy using a proportionality rule for dose adjustment, based on the trough concentration obtained by simulations with RUV (i.e. C_min,RUV_). We also explored an alternative Model-Informed Precision Dosing (MIPD) TDM strategy, with dose adjustment relying on model-derived Bayes estimates of C_min_ or AUC calculated from C_min,RUV_. For all investigated strategies, we targeted the geometric mean of the corresponding therapeutic range and calculated the percentage of patients with IPRED_TRUE_ and AUC_TRUE_ within the target ranges. The doses considered for adjustment were exclusively 100, 200, 300, and 400 mg, in accordance with real-life practicability.

## Results

### Patients data

The initial dataset for popPK model development included 44 patients from centres 1 and 2, three of whom being excluded from the analysis due to unavailable dosage history or inconsistencies between the recorded doses and ISA measurements. Finally, 41 individuals contributed to a total of 196 concentration measurements, with a median of 4 (range: 1–11) samples per patient, for the popPK analysis. The administered ISA daily doses ranged from 100 to 400 mg, with 80% of samples collected during oral administration. Table 1 summarizes the characteristics of the patients included in the study.

**Table 1.** Characteristics of the patients included in the popPK analysis

Characteristics	Median (range) or *n* (%)
Demographics (at baseline)	
Age (years)	61 (20–83)
Body Weight (kg)	70 (32–137)
Male	23 (56)
Height (cm)	171 (150–188)
BMI (kg/m^2^)	24 (12–40)
Laboratory values (all over the study period)	
Creatinine (µmol/L)	86 (16–398)
Total bilirubin (µmol/L)	6 (2–98)
Direct bilirubin (µmol/L)	3 (2–86)
Albumin (g/L)	33 (14–46)
Previous IFI treatment	
Voriconazole	10 (24)
Posaconazole	3 (7)

The centre 3 dataset involved 45 patients taking 200 mg ISA once per day, providing 83 C_min_ measurements with a median of 1 (range: 1–6) per patient.

Among the 27 patients from all three centres with proven/probable IFI and a PK measurement within 50 days from the efficacy outcome evaluation (i.e. date of CT scan), 21 received ISA as first-line therapy or as subsequent-line therapy started within 4 weeks from the beginning of antifungal therapy and had at least 4 weeks of ISA treatment. These patients were included in the PK-efficacy analysis. Therapeutic success (i.e. complete or partial response) was observed in 9 (43%) of them. Liver test disturbances within the PK measurements window were reported for 21 (28%) of the 76 subjects with available information for the PK-toxicity analysis. Treatment discontinuation occurred in 5 of them. Table S1 (available as Supplementary data at *JAC-AMR* Online) presents the characteristics of patients and IFI for PK-efficacy (*n* = 21) and PK-toxicity (*n* = 76) groups.

### Population pharmacokinetic model

A one-compartment model with linear absorption and elimination and IIV assigned only to CL adequately captured ISA PK, with no difference in drug bioavailability (i.e. 100%) after oral or intravenous administrations (ΔOFV = 0.0). The absorption process was best captured by a simple first-order absorption model, with a constant rate (k_a_) fixed at 2.5 h^−1^ to achieve a T_max_ of 2.6 h (Supplementary Materials). Inclusion of IIV on V further improved the model (ΔOFV = −11.0, *P* < 0.01). A mixed error model adequately described RUV. Base model parameters with IIV (CV%) were a CL of 2.2 L/h (35%) and a V of 550 L (56%), with k_a_ fixed at 2.5 h^−1^. Similar parameter estimates were obtained when removing the few observations collected earlier than 6 hours post-dose (data not shown).

After excluding the three concentrations from the dataset of centre 3 with |CWRES|>3, the model predicted all the remaining concentrations with an MPE of 6% (CI95%: −4 to 16) and an RMSE of 62% (Figure S1). The removal of an extremely low concentration reduced the RMSE to 43%, indicating overall good precision in predicting centre 3 data (Supplementary Materials).

**Figure 1.**
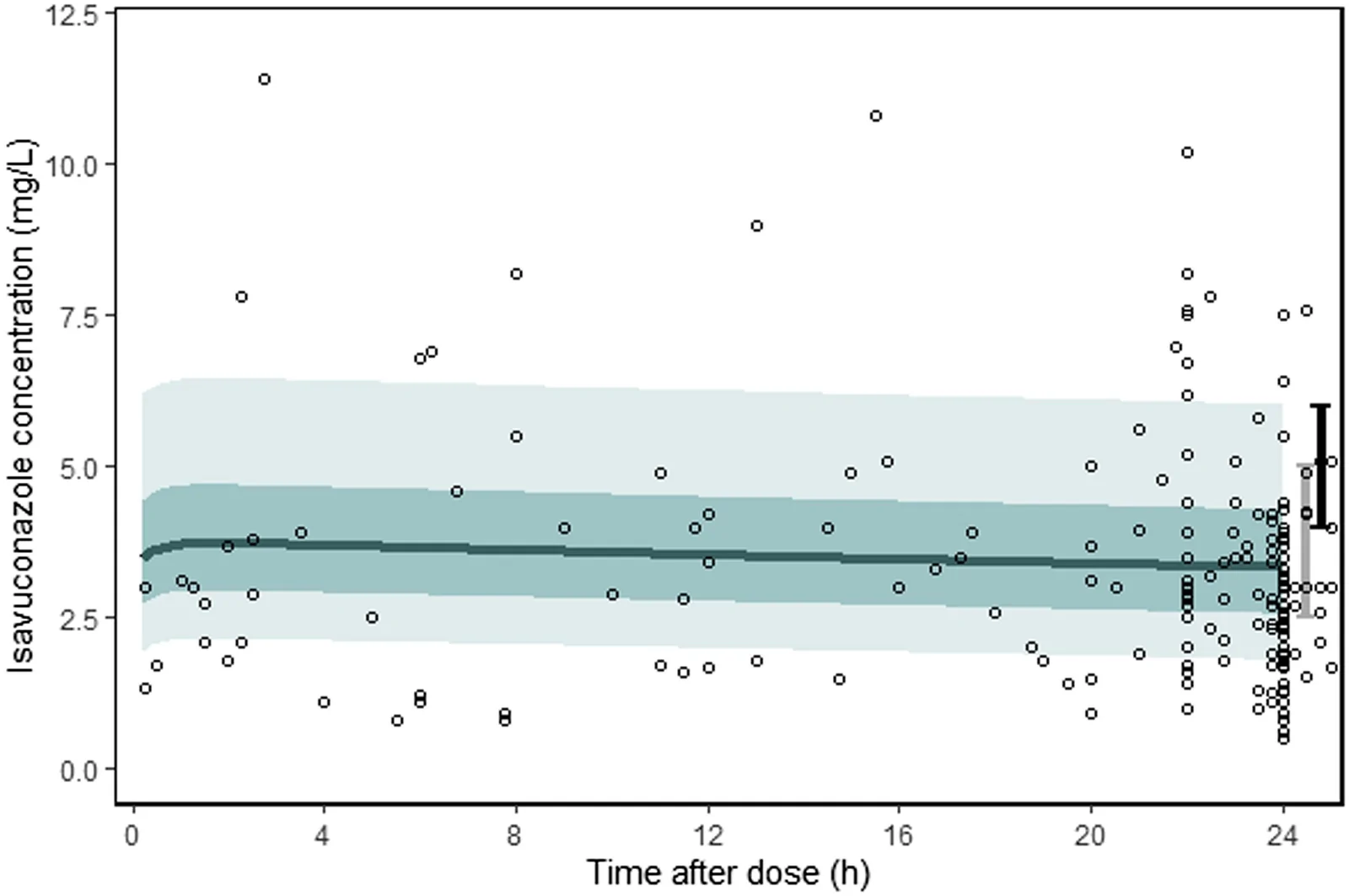
Model-based typical concentration-time profile of isavuconazole under the standard dosage regimen of 200 mg once daily. The continuous line represents the median percentile curve, while the dark and light shaded areas depict the 50% and the 90% prediction intervals generated including only the IIV terms in the model, respectively. The dark gray and the black vertical lines represent the current interval (2.5–5.0 mg/L) and the interval proposed in this study (4.0–6.0 mg/L) as target ranges for trough concentration. Hollow circles represent dose-normalized observed isavuconazole concentrations.

Covariate analyses revealed a significant association between V and BMI, reducing by 61% the variance associated with V from the base model. No other covariate affected ISA disposition. Table 2 shows the final model parameters along with their bootstrap evaluations, which differ by less than 4%, demonstrating the excellent model reliability. The associated GOF plots (Figure S2) and pcVPC (Figure S3) support the model’s adequate predictive performances. Figure 1 presents the typical PK profile of ISA under the standard oral dosage of 200 mg once daily, with percentile ranges illustrating the IIV PK variability in the population.

**Table 2.** Final popPK model parameter estimations along with their bootstrap evaluations

Parameters	Estimate (RSE^a^)	Median bootstrap estimate (CI_95%_^b^)
TVCL (L/h)	2.3 (8%)	2.3 (1.9–2.6)
ω_CL_ (CV%) [η-shrinkage(%)]	37 (15%) [20]	37 (24–48)
TVV (L)	523 (12%)	525 (406–665)
ω_V_ (CV%) [η-shrinkage(%)]	34 (25%) [49]	34 (11–57)
k_a_ (h^−1^)	2.5 FIX	—
Proportional error component model (%)	19 (31%)	19 (7–29)
Additive error component model (mg/L)	0.73 (22%)	0.70 (0.29–0.94)

Final model equations: $CL_{i}=TVCL\cdot e^{\eta_{CL,i}}$ $V_{i}=TVV\cdot \;\frac{BMI_{i}}{25}\cdot e^{\eta_{V,i}}$ $k_{a_{i}}=k_{a}$

where *TVCL* and *TVV* represent the typical population clearance (CL) and volume of distribution (V), respectively; *k_a_* the absorption rate constant; *CL_i_*, *V_i_*, and *k_ai_* the individual *i* parameters; *η_CLi_* and *η_Vi_* the individual random effect terms for *CL* and *V*, respectively, arising from normal distributions centred on 0 and with variances *ω_CL_^2^* and *ω_V_^2^*; *BMI_i_*, the individual *i* BMI.

^a^RSE: relative standard error (SE) calculated as SE/estimate with SE directly retrieved in NONMEM.

^b^CI_95%_: bootstrap 95% confidence interval.

### Exploratory pharmacokinetic-pharmacodynamic analyses

**Figure 2.**
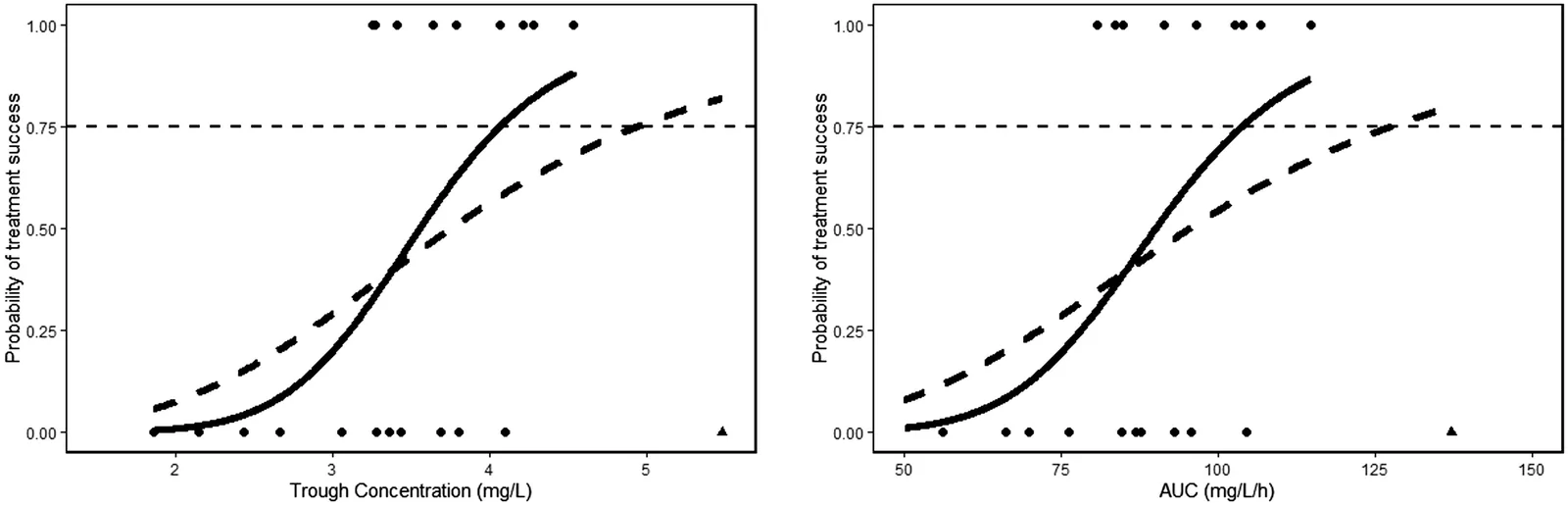
Probability of treatment success versus trough concentrations (left panel) and AUC (right panel). The filled circles and triangles represent the model-predicted individual trough concentrations (left panel) and the AUC (right panel), while the dashed and solid line shows the fitted logistic regression curve obtained using the complete dataset or after removal of the outlier data (triangles). The dashed line indicates the probability of success at 0.75.

Median (range) model-derived Bayes estimates of C_min_ were 3.8 (3.2–4.5) mg/L in patients who recovered from infection and 3.3 (1.9–5.5) mg/L in the others. For the same groups, AUC estimates were 96 (81–115) mg/L/h and 86 (48–137) mg/L/h, respectively. Logistic regression analyses, detailed in the Supplementary Materials, revealed a trend toward a PK-efficacy relationship (Figure 2), after removal of one outlier patient with extreme C_min_ and AUC values (*P* = 0.06). This patient died from uncontrolled leukaemia, while the IFI (mucormycosis) had been treated by surgery with a complete response on chest CT follow-up. Therefore, death was not attributed to IFI in this case. Similar trends were obtained after removing the data from centre 3 (Supplementary Materials).

Patients with liver test disturbances had a median C_min_ of 3.3 (0.3–6.4) mg/L and an AUC of 82 (9–163) mg/L/h, while the subgroup of individuals who subsequently interrupted treatment had a median C_min_ of 2.9 (2.6–6.4) mg/L and an AUC of 73 (64–163) mg/L/h. The respective values were 2.9 (0.5–5.2) mg/L and 74 (13–132) mg/L/h in patients without liver dysfunction. We observed no association in the PK-toxicity analyses (*P* > 0.5).

Thus, our observations do not provide any strong evidence for changing the proposed TDM thresholds.

### Model-based simulations and virtual TDM study

Model-based simulations showed that about 63% of the virtual patients initially achieved the recommended C_min_ target of 2.5 to 5.0 mg/L, and 83% had an AUC_TRUE_ within the AUC range of 60–233 mg/L/h, under a 200 mg once-daily regimen.

Simple TDM and MIPD strategies based on C_min_ target increased similarly these percentages up to 73% and 75% for IPRED_TRUE_, respectively, and both up to 88% for AUC_TRUE_. When the adjustments were performed according to AUC target of 60–233 mg/L/h, 67% of virtual individuals had an IPRED_TRUE_ within the C_min_ target of 2.5–5.0 mg/L and 89% the AUC_TRUE_ within the corresponding target.

## Discussion

This study aimed to characterize ISA popPK in a real-life cohort of patients treated for IFI at three university hospitals in Switzerland. A one-compartment model with first-order absorption and linear elimination adequately described ISA PK, which displays moderate variability. Our parameter estimates are globally comparable with those reported in previously published studies, even those using two-compartment models.^30,46^ The association between V and BMI, as well as the lack of any other clinically relevant PK parameter-covariate relationships, align with previous findings from Phase 1 and Phase 3 trials and patients cohorts.^9,22,27,47^ A first-order absorption process with fixed k_a_ to 2.5 h^−1^ allowed capturing ISA T_max_ adequately, while other complex models did not improve data description.

The clinical follow-up of the patients allowed us to explore the relationships between ISA PK and PD. A trend was observed between C_min_ or AUC and the probability of treatment success, but did not reach statistical significance. Conversely, our analyses did not reveal any association or trend between exposure and toxicity, as also reported by others.^48^ Further studies remain warranted to define a possible TDM target range.

Model-based simulations show that almost half of the virtual subjects receiving ISA standard dosage will not achieve the current C_min_ therapeutic target range of 2.5–5 mg/L, with most of them being underdosed. Similar findings have been reported in real-life cohorts with limited numbers of patients using alternative ISA targets (1–4 mg/L, 2–4 mg/L, or 2–4.5 mg/L), with the highest proportion of individuals exhibiting insufficient ISA exposure when applying more restrictive target ranges.^22,30,47–49^ Because of the small size of RUV error, simple TDM or MIPD dosage adjustment based on C_min_ provided roughly similar results and increased the number of virtual individuals within the target, as also reported in a study performed in a small cohort of patients (*n* = 24), thus supporting the benefit of TDM for optimizing ISA treatment.^30^ The relatively narrow target range, together with the coarse choice of clinically applicable doses, is the main reason why TDM only brought 73% to 75% of simulated patients within the therapeutic range. Having 50 mg capsules would allow for more precise dosage adjustment. A previous popPK analysis based on hospitalized patients drew a different conclusion on the usefulness of ISA TDM, with model-based simulations showing C_min_ > 1 mg/L in almost all virtual subjects receiving a 200 mg maintenance dose.^27^ However, the use of a lower bound of the target range probably explains the higher proportion of patients considered correctly dosed in this study. Meanwhile, both model-based simulations show a low risk of having C_min_ above the upper target values, therefore ensuring patient safety.

A recent study explored the use of individual model-predicted AUC as a marker of ISA exposure, and found that real-life patients exhibited lower drug exposure compared to those enrolled in clinical trials.^49^ Our model-based simulations demonstrate MIPD based on AUC and C_min_ to perform equally well in increasing the number of patients within the target. The long half-life of ISA makes C_min_ highly correlated with AUC at steady state and thus a convenient marker of drug exposure.

We acknowledge several limitations of our study. First, its retrospective design and the sparseness of TDM data, with a lack of exact last dosing and sampling times for centre 3 observations, may introduce some margin of error in the data. However, our PK parameter estimates are closely aligned with those previously reported, which supports our findings. Second, some PK-PD relationships may have remained undetected due to the limited number of patients enrolled. Furthermore, we could not refine the PK-PD analysis by integrating albumin-corrected C_min_ values, whereas variable and saturable protein binding is known to substantially impact ISA exposure-response relationship.^50^ On the other hand, our results are consistent with those of other studies reporting the absence of clinically relevant PK-toxicity associations. Our toxicity analysis focused essentially on liver test disturbances, a well-known adverse effect of triazoles.^51^ ISA is overall well-tolerated with few other reported side effects.^52^ In our study, only one patient interrupted ISA therapy because of potentially related adverse effects (skin rash and gastrointestinal disturbance).^34^ C_min_ values in this case were within the normal range.

In conclusion, this study suggests that TDM may be beneficial to optimize ISA therapy, considering that target therapeutic concentrations were not achieved in a high proportion of patients. Although our PK-PD analysis showed a trend for an association between C_min_ or AUC and outcomes, further prospective investigations in properly sized cohorts remain warranted to confirm these results.

## Supplementary Material

dlag071_Supplementary_Data

## Supplementary data

Figures S1 to S4 and Table S1 are available as Supplementary data at *JAC-AMR* Online.
