# Correction to: Characterization of isavuconazole pharmacokinetics and pharmacodynamics in a real-life cohort

**DOI:** 10.1093/jacamr/dlag214

**Published:** 2026-09-29

This is a correction to: Monia Guidi, Jade Couchepin, Ilana Reinhold, Ilona Kronig, Dionysios Neofytos, Peter Werner Schreiber, Pascal André, Thierry Buclin, Frederic Lamoth, on behalf of the Fungal Infection Network of Switzerland (FUNGINOS), Characterization of isavuconazole pharmacokinetics and pharmacodynamics in a real-life cohort, *JAC-Antimicrobial Resistance*, Volume 8, Issue 3, June 2026, dlag071, https://doi.org/10.1093/jacamr/dlag071

In the originally published version of this manuscript, there was an error in the affiliations listed for co-author Peter Werner Schreiber.

Peter Werner Schreiber's affiliations have been corrected to read:

Department of Infectious Diseases and Hospital Epidemiology, University Hospital of Zurich and University of Zurich, Zurich, Switzerland.

Department of Infectious Diseases and Infection Prevention, Lucerne Cantonal Hospital, Lucerne, Switzerland

Instead of:

Department of Infectious Diseases and Hospital Epidemiology, University Hospital of Zurich and University of Zurich, Zurich, Switzerland.

University of Cologne, Faculty of Medicine and University Hospital Cologne, Department I of Internal Medicine, Excellence Center for Medical Mycology, Cologne.

Subsequent affiliations have been re-numbered accordingly.

