# Supplementary material for: Characterization of isavuconazole pharmacokinetics and pharmacodynamics in a real-life cohort
Source: JAC Antimicrob Resist. 2026 May 5;8(3):dlag071. doi: 10.1093/jacamr/dlag071 (PMC13138873; doi:10.1093/jacamr/dlag071)
Supplement: dlag071_Supplementary_Data [file dlag071_supplementary_data.docx]

**Table S1:** Characteristics of patients included in the PK-efficacy and PK-toxicity analyses.

|  | **PK-efficacy (n=21)** | **PK-toxicity (n=76)** |
| --- | --- | --- |
| Demographic data  Age  Male / Female | 56 (23 – 75)  13 (62) / 8 (38) | 59 (20 – 83)  49 (64) / 27 (36) |
| Underlying diseases  Hematologic cancer  Other | 12 (57)  9 (43) | 55 (72)  21 (28) |
| IFI type  Invasive aspergillosis  Invasive mucormycosis  Other | 11 (52)  6 (29)  4 (19)^1^ | 25 (33)  8 (11)  43 (57)^2^ |
| IFI classification  Proven  Probable  Possible  No IFI | 16 (76)  5 (24)  -  - | 26 (34)  11 (14)  30 (39)  9 (12) |
| IFI site  Lung only  Lung + other organ(s)  Extra-pulmonary site only  No documented site | 12 (57)  6 (29)  3 (14)  - | 56 (74)  10 (13)  8 (11)  2 (3) |
| ISA therapy  First line  Subsequent line  Duration | 9 (43)  12 (57)  126 (28 – 439) | 35 (46)  41 (54)  95 (7 – 953) |

Results are expressed as number of cases (percentage) for proportions and median (range) for continuous variables.

PK: pharmacokinetic, IFI: invasive fungal infection, ISA: isavuconazole.

^1^ Other include: mixed invasive aspergillosis and mucormycosis (n=1), invasive scedosporiosis (n=1), *Conidiobolus* invasive infection (n=1), proven IFI without microbiological documentation (histopathology only, n=1).

^2^ Other include: mixed invasive aspergillosis and mucormycosis (n=1), documented IFI other than invasive aspergillosis or mucormycosis (n=4), possible or suspected IFI without microbiological documentation (n=38).

**Population pharmacokinetic model**

The Weibull model proposed by Desai et al^1^ did not provide a better fit of the data (ΔAIC = +4.0), nor did the use of a two-compartment model (ΔOFV = -5.6, p>0.05) compared to a simple linear absorption model with ka fixed at 2.5 h^-1^.

Forward covariate inclusion step revealed an association between V and body size markers integrated using allometric scaling with power fixed at 1 (ΔAIC = -7.0 for BW and ΔAIC = -6.9 for BMI), along with a linear impact of CLcr on CL (ΔOFV = -5.6, p<0.05). No other factors were significantly associated with ISA PK (ΔOFV < -2.6, p<0.05). Multivariate covariate analyses followed by backwards deletion step led to discarding the effect of CLcr on CL, and retaining only the effect of BMI on V, with BMI preferred over BW as body size marker.

**Figure S1:** Goodness of fit plots of the base isavuconazole popPK model applied to the external validation dataset (centre 3 data) after exclusion of the three concentrations with |CWRES]>3.


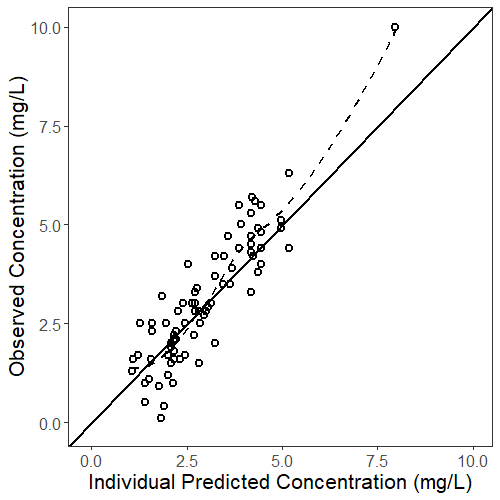

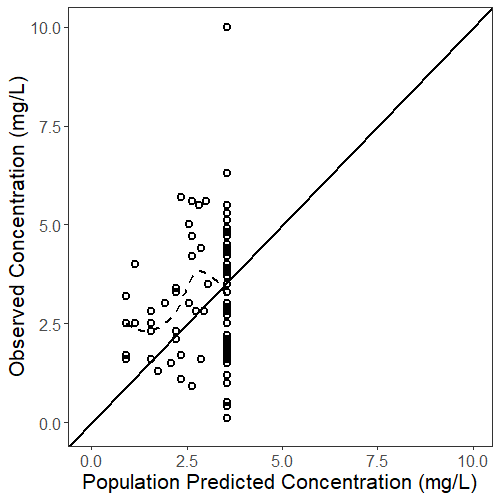


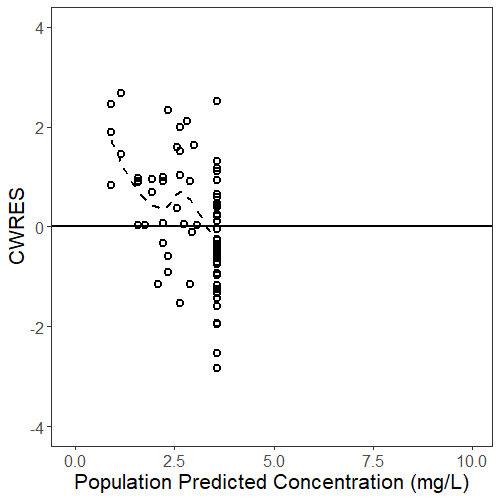

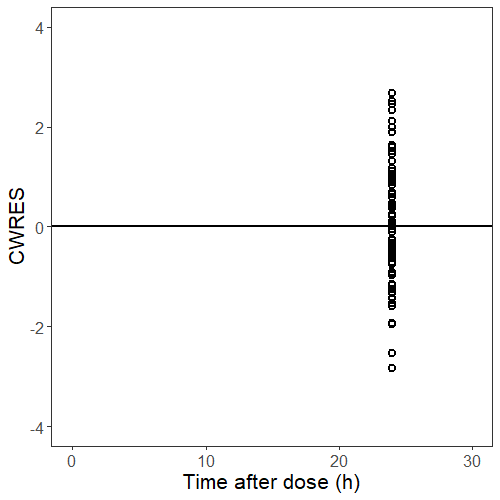


We identified a very low concentration measurement of 0.1 mg/L for one patient enrolled in centre 3. The exclusion of this concentration reduced the MPE (CI95%) to -3% (CI95%: -10 to 5) and the RMSE to 43%, which indicates overall a non-significant bias and good precision in predicting the centre 3 concentrations by the population PK model developed on centres 1 and 2 data.

**Figure S2:** Goodness of fit plots of the final isavuconazole popPK model (centres 1 and 2 data).


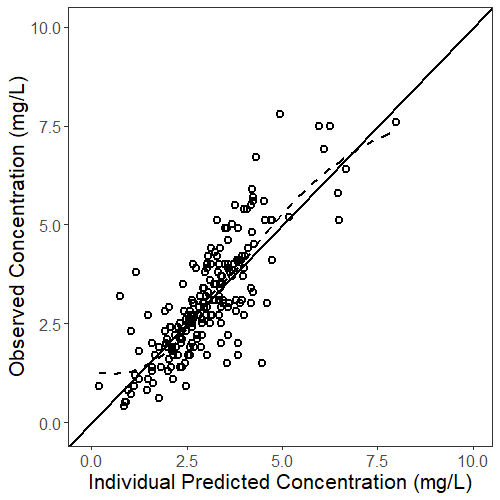

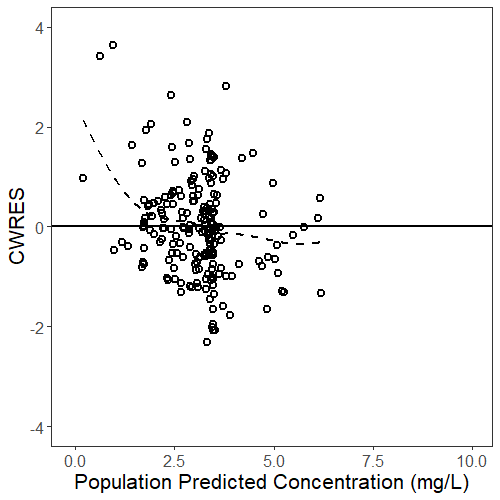


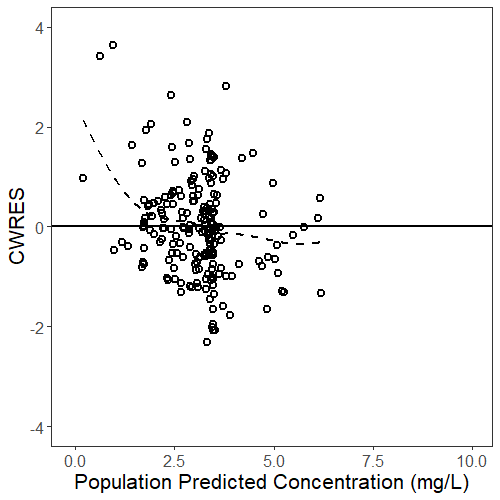

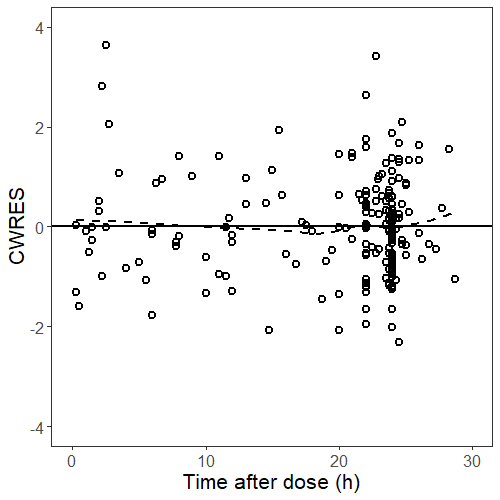


**Figure S3**: Prediction-corrected Visual Predictive Checks (pcVPC) of the final isavuconazole popPK model. The dots represent the prediction-corrected observations, summarized by the median (solid line), 5^th^ and 95^th^ percentiles (dashed lines). The shaded areas indicate the corresponding model-predicted 90% confidence intervals.


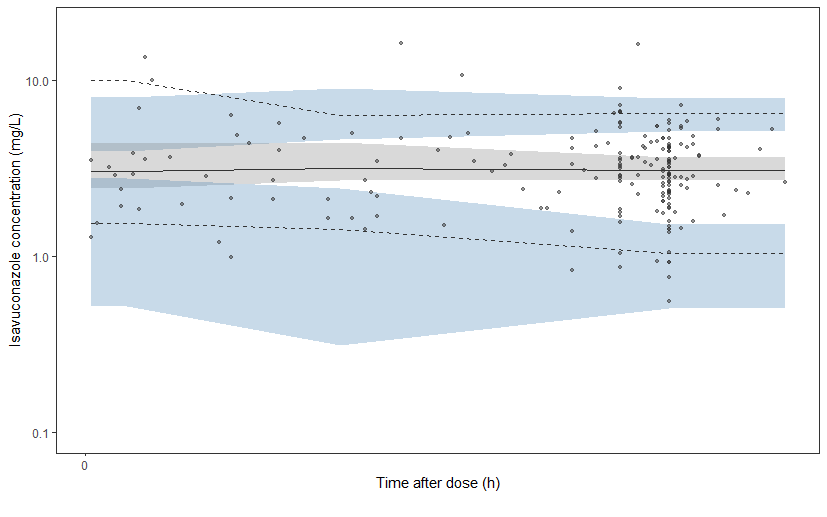


**Exploratory Pharmacokinetic-Pharmacodynamic analyses**

Logistic regression analyses did not reveal a significant association between log-transformed C_min_ or AUC and the probability of success (p=0.11 and 0.14, respectively), the probability of experiencing hepatic test disturbances (p=0.86 and 0.94, respectively) or consequent treatment interruption (p=0.62 and 0.62, respectively).

In the PK-efficacy analysis, we excluded the patient with C_min_ of 5.5 mg/L and AUC of 137 mg/L/h, who died from uncontrolled leukemia.

The logistic regression analysis on the patients from centres 1 and 2 (n=13, after removal of the outlying patient with very high C_min_ and AUC values) did not identify a statistically significant association between the outcomes and the exposure markers (p=0.2 for both C_min_ and AUC, Figure S4).

**Figure S4:** Probability of treatment success versus trough concentrations (left panel) and AUC (right panel). The filled circles and triangles represent the model-predicted individual trough concentrations (left panel) and the AUC (right panel), while the dashed and solid line shows the fitted logistic regression curve obtained using the complete dataset or after removal of the outlier data (triangles). The dashed line indicates the probability of success at 0.75.


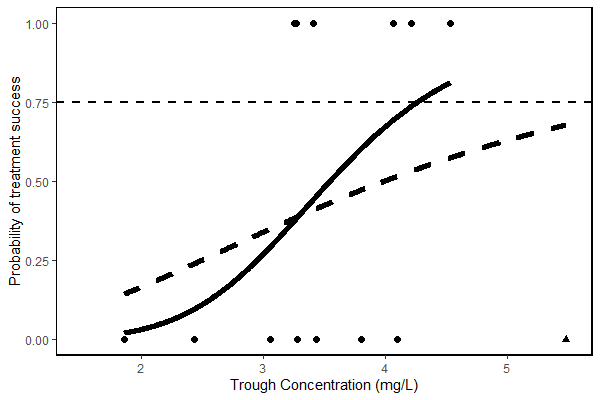

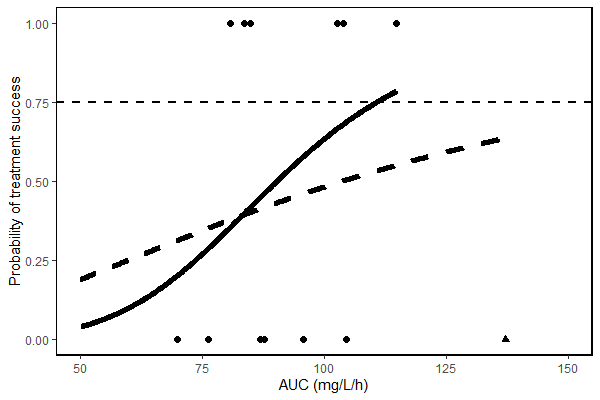


However, these results indicate a probability of success exceeding 0.75 for C_min_ and AUC very close to what has been identified as thresholds in the exploratory PK-efficacy analysis including the centre 3 data.

***References***

1. Desai, A., L. Kovanda, D. Kowalski, Q. Lu, R. Townsend, and P.L. Bonate, *Population Pharmacokinetics of Isavuconazole from Phase 1 and Phase 3 (SECURE) Trials in Adults and Target Attainment in Patients with Invasive Infections Due to Aspergillus and Other Filamentous Fungi.* Antimicrob Agents Chemother, 2016. **60**(9): p. 5483-91.
